# School emphasis on academic success: the role of principal qualifications

**DOI:** 10.3389/fpsyg.2024.1288174

**Published:** 2024-07-29

**Authors:** Georgios Sideridis, Mohammed Alghamdi

**Affiliations:** ^1^Boston Children’s Hospital, Harvard Medical School, Boston, MA, United States; ^2^National and Kapodistrian University of Athens, Athens, Greece; ^3^Department of Self-Development Skills (SDS), King Saud University, Riyadh, Saudi Arabia

**Keywords:** principals, attitudes towards success, TIMSS 2019, MIMIC model, Transformational leadership

## Abstract

The purpose of the present study was to relate a principal’s qualifications with a school’s emphasis on academic success. Participants were *n* = 206 principals of respective schools in Saudi Arabia that took part in the study as a function of the TIMSS-2019 assessment. Principals were administered the eleven-item “School Emphasis on Academic Success” scale. A binary covariate defining low and high principal qualifications was computed. The Multiple Indicators Multiple Causes (MIMIC) model was involved with the latent means of both a general and three specific factors being regressed on the covariate to evaluate latent mean differences across differentially qualified principals. Furthermore, each one of the instrument’s indicators was regressed on the principal covariate to evaluate the presence of Differential Item Functioning (DIF) or in other words additional effects due to item content. Results indicated a significant omnibus effect for the general factor only, with highly qualified principals holding significantly more positive beliefs about how parents, teachers, and students feel about their school’s emphasis on academic success. Further analyses at the item level indicated that “teacher expectations” were the single item presenting a DIF effect with highly qualified principals having stronger beliefs about their teacher’s expectations of student success over and above the latent factor mean. Results are discussed on how they inform educational policy and practice.

## 1 Introduction

Principals play a key role in establishing an environment in schools that supports learning and success (Deal and Peterson, 2003; Leithwood et al., 2008; May et al., 2012; Thapa et al., 2013; Reid, 2020). Knight Abowitz (2019) added that very few professions are as challenging as that of the school principal due to a large load of diverse responsibilities and roles (Rousmaniere, 2013). The difficulty stems from the placement of the principal as an intermediary between schools and districts to serve both while having to navigate between conflicting policies, interests, and community demands. Undoubtedly, empirical evidence has confirmed the role of principals in (a) creating a warm, caring, and supportive school community (Louis et al., 2016; Sørensen and Robertson, 2017, 2018; Ryu et al., 2020) (b) cultivating a positive school climate (Leithwood and Jantzi, 1999; Marks and Printy, 2003; Hoy et al., 2006; MacNeil et al., 2009; Leithwood and Sun, 2012), (c) encouraging collaboration between teachers and parents (Barr and Saltmarsh, 2014), (d) managing conflicts (Ghaffar et al., 2012), (e) assessing and regulating own and other’s emotions (Cunningham, 1983; Litt and Turk, 1985), (f) promote motivation and self-satisfaction by fostering professional relationships (Bird et al., 2009; Cherkowski, 2012; Louis and Murphy, 2017), and (g) increase teacher retention (Boyd et al., 2011), among other.

### 1.1 Transformational leadership theory: description and empirical findings

The above outcomes are linked to the transformational leadership theory which posits that effective leaders yield results through inspiring and motivating their staff members to unite in a common vision, promoting creativity, and enabling personal growth (Burns, 1978; Bass, 1985). In the scope of this theory, school principals have an indirect rather than direct role in shaping the school culture and climate, improving relationships, creating partnerships, and enhancing resources (Hallinger and Heck, 1996; Giles, 2006; Korkmaz, 2007; Nettles and Herrington, 2007; Shatzer et al., 2014). Principals who exhibit leadership talents such as trust building, motivation, communication, collaboration, and teamwork can develop a learning environment where innovative culture, collaboration, and continued improvement can thrive (Finnigan, 2010). Studies have shown that transformational principals impact teachers to engage in innovative practices, commit, grow, and realize goals (Murphy et al., 2007; Corcoran et al., 2013; Murphy et al., 2013), ultimately promoting their school success. For example, Bowers et al. (2017) reported that teachers in schools with low levels of leadership reported employing fewer and less effective evaluations of their instruction and reported more professional isolation. Teacher’s views on leadership also deviated markedly from those of their principals in relation to structuring time and in investing in school resources for student learning (Bowers et al., 2017). Liu (2021) reported that high levels of transformational leadership had a positive impact on teacher’s collective efficacy. Vecchio et al. (2008) found that transactional leadership is associated with enhanced teacher performance and satisfaction with the effects being more pronounced for performance.

Thus, school quality is tied to a great degree to principal qualifications, skills, and competencies (Hallinger and Heck, 1996; Hallinger, 2011; Hallinger et al., 2019). Research has supported that principal trust is indirectly associated with increased teacher efficacy and student academic outcomes (Çoban et al., 2023). Principal’s focus on instruction has also been linked to student gains in learning outcomes (Robinson et al., 2008; Bryk et al., 2010; Sebastian et al., 2017; Goddard et al., 2019). Principal’s leadership and commitment have been linked to teacher commitment (Hallinger and Lu, 2014; Hallinger et al., 2017; Al-Mahdy et al., 2018) and teacher efficacy (Cansoy et al., 2020). Interestingly, as Al Sadaawi (2010) reported, quality is not equivalent to quantity in terms of the amount of expenditure. In Saudi Arabia, although ¼ of the government spending goes to education, students’ academic achievement remains at low levels in relation to international standards. For example, based on the 2015 TIMSS data the achievement in mathematics of 8th graders in Saudi Arabia was ranked last among 39 countries (Mullis et al., 2016) and the trend was consistent in 2019 as well with the students having the 3d worst achievement across all countries. New governmental policies have been introduced to decentralize authority towards principals, improve classroom practice, and hire better teachers (Alshaya, 2011). Aburizaizah et al. (2019) added that principal leadership in Saudi Arabia involving principal’s efforts to enrich the academic climate, provide teacher incentives, and enhance instructional resources were associated with improvements in mathematics achievement over time, particularly for boys. Although these findings were encouraging, findings against empowering principals have also been reported (Paletta, 2014; Fuller, 2015). Thus, with regard to the role of principals, the jury is still out.

Given the critical role of school leaders in educational reform, the Organization for Economic Cooperation and Development (OECD) created a framework to define the role of school leaders (Berkovich and Benoliel, 2021) to identify “ideal” school leaders. Based on OECD school leaders are responsible for a school’s strategic direction by developing and executing a plan of action in line with national and international standards. An emphasis on successful management is denoted by their ability to motivate unmotivated employees, monitor implemented practices, create a harmonious environment, and engage in scientifically guided practices (Berkovich and Bogler, 2019).

### 1.2 School principals as creators of self-fulfilling prophecies

School principals’ attitudes and behaviors have a direct influence on teachers through influencing expectations (McKown and Weinstein, 2008). By cultivating a climate of positivism, respect, and value, a sense of collective efficacy can be developed (Cansoy et al., 2020), which can then lead to the creation of a self-fulfilling prophecy toward success (Marzano et al., 2003). In the context of education, a self-fulfilling prophecy can occur when a teacher or principal has a preconceived notion about a student’s abilities or potential and unconsciously treats that student in a way that confirms those beliefs. In other words, the belief and anticipation of a specific outcome can bring about its realization. For example, if a principal believes that a student is not capable of academic success, they may unconsciously convey negative messages to teachers who may provide less support and encouragement to that student, leading the student to perform poorly. The poor performance then confirms the principal’s original belief, which is also transferred to the teacher, and the vicious cycle of low expectations and low achievement continues. Similarly, if a principal believes that a certain group of students (e.g., students from low-income families) are not capable of academic success, or that his school’s lack of resources cannot be overcome (Prabhakar et al., 2022), they may unconsciously reinforce these original beliefs of lower aptitude and lower achievement. Transformational leaders promote high expectations and instill a sense of efficacy in their teachers to exert excessive effort and be perseverant to obstacles and challenges (McKown and Weinstein, 2008). Therefore, school principals need to have positive views about their teachers’ competencies and skills, students’ potential, and parent engagement so that all together can reinforce a positive self-fulfilling prophecy.

In the present study, we examine the presence of transformational leadership by classifying leaders based on their education, experience, and leadership training. We extend past research by examining how transformational leadership from principals influences their expectations on the roles of teachers, parents, and students towards achieving academic success. We hypothesize that principals with high levels of transactional leadership would have significantly more positive beliefs regarding parent, teacher, and student perceptions of their school’s emphasis on academic success.

## 2 Materials and methods

### 2.1 Participants and procedures

Participants were 209 school principals who participated in the TIMSS 2019 and contributed data for the 8^th^-grade cohort in Saudi Arabia. Among them, n = 206 provided complete data and comprised the final sample. The mean amount of time being principals was 8.5 years (SD = 7.818) and for the specific school, they reported serving an average of 4.81 years (SD = 5.135). Most of the principals held a bachelor’s degree (N = 180, 87.8%), 15 held a master’s degree (7.3%), 2 held a doctorate (1%) and 8 did not have a Bachelor’s degree (3.9%). More information on the data can be found in the source.^1^

Participants came from schools of which 63.2% were located in urban areas, 4.5% in suburban areas, 17.9% in medium-sized cities or large towns, 11.9% in small towns or villages, and 2.5% in remote, rural areas. Furthermore, 54% were classified as affluent as per the TIMSS socioeconomic measure, and 15.3% as disadvantaged. The remaining 30.7% of the schools were considered to be in the average range. The schools represented all 13 regions of the Saudi Arabia country.

### 2.2 Measures

#### 2.2.1 School emphasis on academic success

This 11-item scale on the TIMSS 2019 study involves the beliefs of principals regarding how parents, teachers, and students perceive that their school has an emphasis on academic success. The content of the scale relates to three components, principals’ views on (a) teacher perceptions, (b) parent perceptions, and, (c) student perceptions on the extent to which their school is focused on academic success. The instrument involves a 5-point scale ranging from Very High to Very low (i.e., Very high, high, medium, low, and very low). The developers provide a non-linear categorical system of three levels, namely, very high emphasis, high emphasis, and medium emphasis by grouping the response options. In the present study, however, we tested rather than assumed the developer’s factor structure of the instrument. Thus, we tested for the presence of a unidimensional latent variable versus a 3-dimensions structure, and the bifactor model using Confirmatory Factor Analysis (CFA), described next (Gignac and Watkins, 2013). Our goal, after concluding on the optimal simple structure was to estimate factor scores so that between groups evaluations would be based on the most accurate latent trait estimates. Items responses were recoded so that higher scores indicate a high emphasis on academic success.

#### 2.2.2 Principal qualifications

Three criteria comprised the measurement of principal qualifications, namely, certificates on educational leadership, education, and experience. Regarding certification, three dichotomous items on the TIMSS contributed information on the presence of additional qualifications in the form of certificates or licenses, related to educational leadership. Principals with graduate degrees in the form of a master’s or Ph.D. degrees were also placed in the highly qualified group. A last distinction classified highly qualified principals as those who had above the median years of experience within their specific school [*F*(1, 200) = 4.179, *p* = 0.042]. Thus, the criteria comprising the highly qualified principal group fit within the transformational leadership theory criteria of (a) enhanced experience in leadership roles, (b) education and training to enhance competency in educational leadership, and (c) soft skills developed through academic degrees and training (Filipczak et al., 1996; Wilson Heenan et al., 2023). By using the above criteria, 55 participants (26.3%) were deemed highly qualified, and 154 (73.7%) as belonging to a low-qualification group. This grouping variable was further utilized to contrast principal beliefs across domains of the emphasis on the academic success scale.

## 3 Data analyses

### 3.1 Confirmatory factor analysis: testing an optimal simple structure

Data were analyzed using Confirmatory Factor Analysis (CFA) and Mplus 8.10 (Mutheen and Mutheen, 1998–2011). Due to the categorical (ordered) nature of the data and the likelihood that the assumption of multivariate normality would not be met (Wang and Wang, 2012; Brown, 2015), the mean and variance-adjusted weighted least squares (WLSMV) estimator was employed (Muthén et al., 1997). The CFA model was employed and tested three competing models, a unidimensional, a 3-factor correlated model, and a bifactor model. Besides evidence on factorial validity, estimates of internal consistency reliability using the omega coefficient (Raykov, 1997) were also employed. Model fit was judged using the *Root Mean Squared Error of Approximation* (RMSEA), the *Comparative Fit Index* (CFI), and the *Tucker-Lewis Index* (TLI), also termed the Non-Normed Fit Index (NNFI). For the RMSEA, values less than 0.08 point to an acceptable model fit, with values <0.05 suggesting an excellent or exact fit of the data to the model (Hu and Bentler, 1998, 1999). For CFI and TLI estimates over 0.90 signal acceptable model fit (with values >0.95 being ideal; Hu and Bentler, 1999).

### 3.2 Multiple indicators multiple causes model (MIMIC)

Following confirmation of the hypothesized simple structure, a Multiple Indicators Multiple Causes (MIMIC) model was applied to evaluate whether the principal’s qualifications are related to both the latent mean of the School’s Emphasis on Success, as well as each indicator of that latent variable. The MIMIC model represents a special form of structural equation modeling (SEM) in that it integrates hypothetical causal variables (i.e., covariates) with the confirmatory factor analysis model (MacIntosh and Hashim, 2003; Finch, 2005; Chun et al., 2016). The MIMIC model incorporates a measurement model and a structural model. The first examines construct validity by confirming (or not) the relationship between measured indicators and latent constructs. The second evaluates the relationship of the covariate(s) on levels of the latent variable(s) as well as each indicator of the latent construct (Brown, 2015). The model’s advantage is the simultaneous evaluation of both measured and structural models as the estimated parameters account for the effects of covariates on latent and measured indicators (Muthen et al., 1991; Brown, 2015) and the lower requirements on sample size (French and Finch, 2008).

As mentioned above, the MIMIC model can also include direct effects of the covariates on indicators, holding the latent variables constant and/or estimating indirect effects via the factor, as in mediational models. These direct paths can examine possible differential responses of the item at different levels of the covariate (in the present study, principal qualifications), which is termed Differential Item Functioning (DIF) (Dorans and Holland, 1993). The MIMIC model tests the probability that item u_j_ that belongs to factor η_i_ and receives a direct effect from a categorical x_i_ (e.g., binary variable defining principal qualifications) has a response probability of 1 as shown below (Mutheen, 1989):


(1) $$u_{ij}=\lambda_{j}\eta_{i}+\kappa_{j}x_{i}+\varepsilon_{ij}$$

with λ being the factor loading of item u_j_ on factor η with a mean of zero, κ_j_ being the effect of the covariate on item u_j_ at values x_i_. The probability of a correct response is then estimated as follows:


(2) $$P\left(u_{ij}=1|\eta_{i},x_{i}\right)=1-F\left[\tau_{j}-\lambda_{j}\eta_{i}-\kappa_{j}x_{i}\right)\frac{1}{\sqrt{\theta_{jj}}}],$$

**Figure 1:**
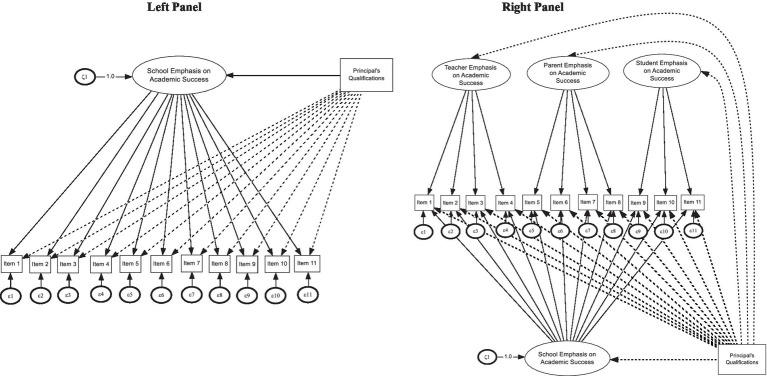
Base MIMIC model assuming a unidimensional structure and one covariate (left panel) versus a model assuming teacher, parent, and student latent variables regressed on principal qualifications (right panel).

With θ_jj_ being the item residual variance, τ_j_ the item threshold, λ_j_ the factor loading, η_i_ the factor being estimated, κ_j_ the effect of the covariate on the item, and *F* the normal distribution function (Muthén et al., 1993). Measurement errors were adjusted using two methods, (a) by utilizing the robust Maximum Likelihood methodology (MLR) and (b) by including school as a clustering variable, thus accounting for school-level influences. A visual of a simple MIMIC model is shown in Figure 1 (upper panel).

Power for the MIMIC model was estimated using a Monte Carlo simulation estimated in Mplus 8.10 rather than using conventions of the RMSEA (MacCallum et al., 1996). A one-factor model with 11 indicators was simulated with slopes equal to 1 and errors of variance equal to 1 reflecting a unidimensional structure although other competing models were also utilized. Of interest in the simulation, however, was the power of the regression of the latent factor on the grouping variable. This path was set to a standardized estimate of 0.35, being reflective of a small-to-medium effect (between 0.2–0.5, S.D., Cohen, 1992). Results using 1,000 replicated samples of *n* = 200 indicated that the power of the grouping covariate was 99.8% with a coverage of 95.2% across the 1,000 replicated samples. Thus, the power of the MIMIC model was adequate in identifying the effects of principal qualifications on the latent means and/or item indicators.

#### 3.2.1 MIMIC model evaluative stages

Several models were employed to confirm the acceptability of the MIMIC model, after confirming the optimal factor structure of the measure (Woods, 2009; Kim et al., 2011; Woods and Grimm, 2011; Kim and Cao, 2015). The baseline model (M0) involved the optimal model among the competing unidimensional, 3-factor correlated, and bifactor models. The first competing model (M1) included a direct path from the covariate principal’s qualifications on the latent mean(s) of the measure. The acceptability of this path was evaluated using a loglikelihood difference test, which is distributed as a chi-square statistic, reflecting the difference between two nested models. The second model (M2) included, in addition to the direct path of the principal’s qualifications on the latent mean, paths between principal qualifications and all indicators of the School’s emphasis. This model, if not significant, then engages 11 models in which the covariate was linked to each one of the model’s indicators. These models were termed M2a-M2k for each one of the 11 indicators of the latent factor and were contrasted to M1.

## 4 Results

### 4.1 Factorial validity of school’s emphasis on academic success

After fitting a unidimensional structure to the 11 indicators of the TIMSS scale using the WLSMV estimator results indicated an acceptable model fit. Specifically, CFI and TLI were 0.966 and 0.957, respectively. The unstandardized residuals ranged between 9.7 and 13.5% with a mean of 11.6, which is outside acceptable standards, most likely given the modest sample size (as in the estimation of RMSEA, the discrepancy function is divided by sample size). A second competing model involved the estimation of 3-correlated domains as the content was focused specifically on teachers (4 items), parents (4 items), and students (3 items). The 3-factor correlated model provided improved model fit mainly by focusing on the residuals [CFI = 0.959, TLI = 0.945, RMSEA = 0.07]. Model comparison using −2* the loglikelihood indicated a superior model fit of the 3-factor correlated model compared to the unidimensional structure (see Table 1). The third and final model involved estimating a bifactor structure so that a test of the presence of both general and specific domains would be evaluated. The bifactor model also had a good fit and, statistically speaking, was superior to the 3-factor correlated model using a test of difference (see Table 1). The bifactor model pointed to the presence of a general factor with all items loading significantly on the general factor and also three distinct dimensions with all but one item, not loading significantly. Specifically, the 3d item from the teacher domain did not have a significant weight, but all other items contributed significantly to both the general and specific domains. Consequently, a MIMIC model based on the bifactor model was utilized (see Figure 1 lower panel).

**Table 1:** Model comparison for identifying the most optimal factor structure.

Models tested	LL	Npar	scf	Model Comp.	LRTS	dtsc	sLRTS	d.f.	*p*-value
a. Univariate	−1632.61	33	1.01	–	–	–	–	–	–
b. 3-Factor Cor.	−1588.64	36	1.01	Uni vs. 3-factor	87.938	1.067	82.401	3	<0.001
c. Bifactor	−1575.57	44	1.02	3-factor vs. bifactor	26.136	1.062	24.617	8	0.002

LL, Loglikelihood; Npar, number of estimated parameters; scf, scaling correction factor; LRTS = −2 (LL0-LL1); dtsc = (*p*0 * c0 – *p*1*c1)/(*p*0 – *p*1); sLRTS = LRTS/dtsc, which is distributed as a chi-square statistic; df = degrees of freedom, i.e., *p*1–*p*0; *p*-value = reflects the chi-square p-value for the given difference in degrees of freedom; Cor, Correlated.

Further evidence on the psychometrics of the proposed model was provided by omega reliability. Estimates of omega internal consistency reliability were 0.92 for the general factor, 0.78 for the teacher factor, 81 for the parent factor, and 0.75 for the student factor in the optimal bifactor structure.

### 4.2 Principal’s qualifications predicting school’s emphasis on academic success

**Figure 2:**
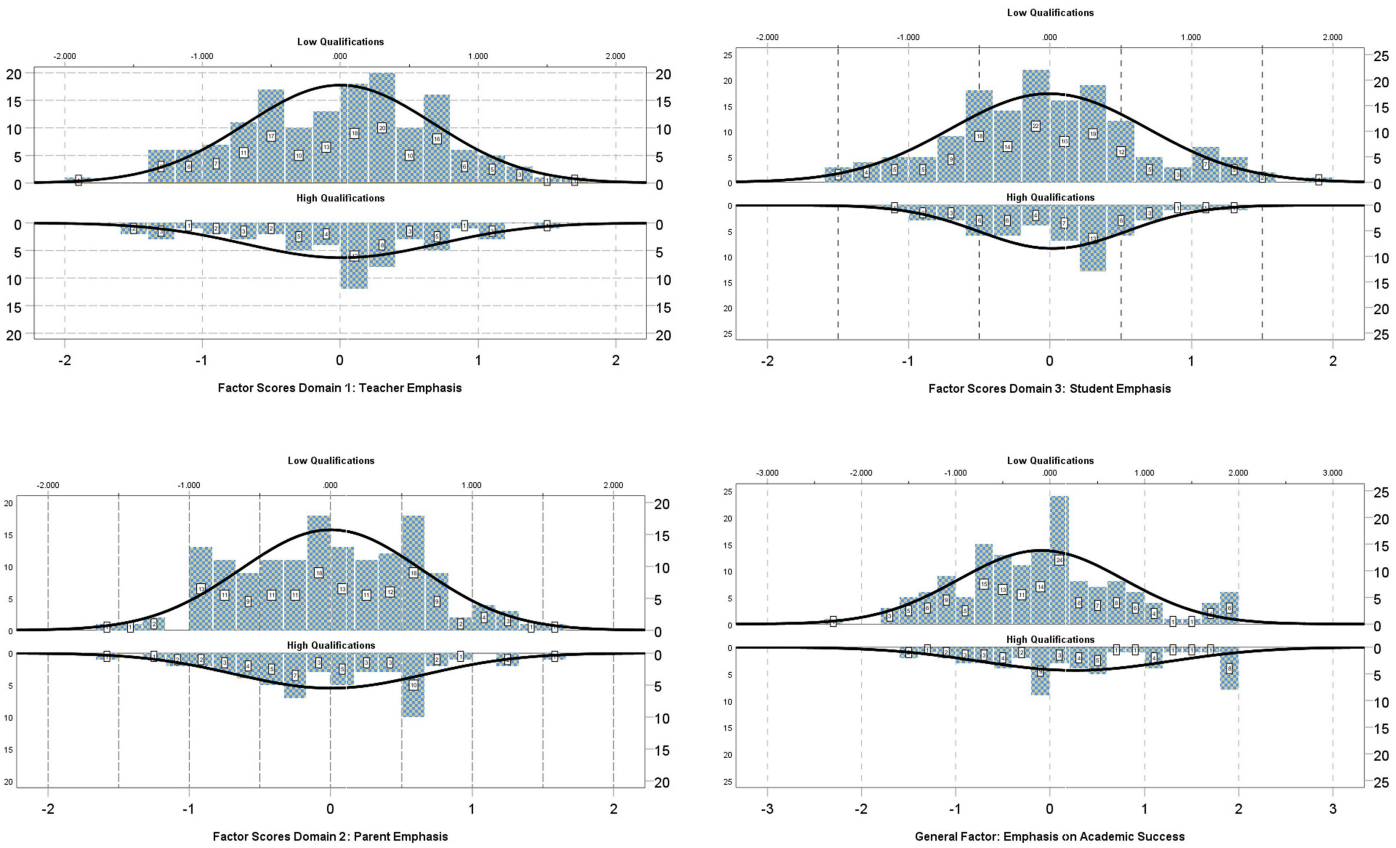
Distribution of factor scores for low and highly qualified principals across domains and general factor.

As shown in Table 2, after specifying a direct path between the principal’s qualifications on the latent means of both the general and specific factors of the School’s Emphasis on Success, model fit was significantly improved [M0 vs. M1: -2*LL = 82.401, *p* < 0.001]. The additional standardized direct effect of the principal’s qualifications on the School’s emphasis on success (*b* = 0.303, *p* = 0.048) suggests that highly qualified principals held stronger beliefs about their school’s emphasis on academic success, overall. There were no significant differences, however, across low and highly qualified principals on the specific domains [b_Teacher Emphasis_ = −0.210, *p* = 0.353; b_Parent Emphasis_ = −0.282, *p* = 0.400, b_Student Emphasis_ = −0.297, *p* = 0.532], with estimates being around the expected amount of measurement error when null hypotheses are supported. Figure 2 displays the distributions of factor scores for each one of the three domains of school emphasis on academic success with the addition of the general factor (containing all items). All figures are split between low-qualified (upper panel) and highly qualified principals (lower panel). As shown by the fitted normal curve, the mean of the latent trait was significantly higher for the general factor only (bottom figure) and the highly qualified principal group compared to the lower qualified group by a 0.3 standard deviation, which reflects a small-to-medium effect size (Cohen, 1992).

**Table 2:** Model comparison for identifying the most optimal MIMIC model towards identifying the role of principal qualification.

Models	LL	Npar	scf	Model Comp.	LRTS	dtsc	sLRTS	d.f.	*p*-value
M0	−1575.570	44	1.022	–	–	–	–	–	–
M1	−1571.015	48	1.013	M0 vs. M1	9.110	0.909	10.019	4	0.040*
M2	−1566.891	59	0.911	M1 vs. M2	8.248	0.467	17.653	11	0.089†
M2c	−1568.743	49	1.023	M1 vs. M2c	4.544	1.532	2.966	1	0.085†

M0 refers to the baseline model. M1 includes a direct effect of the principal’s qualifications on the latent mean. M2, in addition to M1, includes direct paths of the principal’s qualifications across all items of the latent factor. Amongst, models 2a–2k, only the one that showed significant effects was included, i.e., M2c which refers to “Teacher’s expectations of student success.”

Scf = scaling correction factor; Npar = number of estimated parameters; LRTS = −2 (LL0–LL1); dtsc = (*p*0 * c0 – *p*1*c1)/(*p*0 – *p*1); sLRTS = LRTS/dtsc, which is distributed as a chi-square statistic; df = degrees of freedom, i.e., *p*1–*p*0; *p*-value = reflects the chi-square p-value for the given difference in degrees of freedom.

*p < 0.05; **p < 0.01; ***p < 0.001; †*p* < 0.05, one-tailed test.

Model 2 added 10 additional parameters reflecting the direct effects of principal qualifications on each of the 11 indicators’ thresholds of the school’s emphasis on academic success. This model (M2) was not superior to M1 suggesting that the principal’s qualifications did not exert significant effects across all indicators [M1 vs. M2: -2*LL = 17.653, *p* = 0.089], although it trended towards significance. Consequently, a series of models tested the adequacy of direct paths for one indicator at a time using a stepwise iterative procedure. As shown in the table, the only indicator for which significant effects were observed was “Teacher expectations of student success” with the omnibus model fit again trending towards significance [M1 vs. M2c: -2*LL = 2.960, *p* = 0.03]. Similarly, to the global latent mean effect, highly qualified principals held significantly elevated beliefs that their teachers had high expectations of their students’ success (*b* = 0.177, *p* = 0.03). Thus, principals’ dichotomization of qualifications into low and high was associated with significantly different beliefs about their school’s emphasis on academic success, with a particular emphasis on the effects of highly qualified principals on teacher expectations of students’ success.

### 4.3 Examining whether SES confounds the relationship between principal’s qualifications and emphasis on academic success

In TIMSS a composite SES index classifies schools in three levels ranging between disadvantaged and affluent. To test for the moderating role of a school’s SES two analyses were undertaken. First, a 3×2 crosstabulation with SES (disadvantaged-affluent) and principal qualifications (low-high) tested the hypothesis that more qualified teachers were in more affluent schools. This hypothesis was not supported [*χ*^2^ (2) = 2.024, *p* = 0.364]. Furthermore, a 3 × 2 factorial analysis of variance was utilized to examine whether estimated factor scores for each domain as well as the general factor were a function of the moderating effects of SES. Results indicated that none of the 2 × 3 interactions were significant. Consequently, the hypothesis that a third variable behind principal qualifications was SES was not supported.

## 5 Discussion

The purpose of the present study was to relate a principal’s qualifications with their beliefs related to how teachers, parents, and students perceived their school as having a focus on academic success. It is well known that teacher positive expectancies are associated with students’ academic success (Becker, 1952; Brophy, 1983; Rosenthal and Jacobson, 2003; Boer et al., 2018) but they can lead to the creation of self-fulfilling prophecies which can further reinforce and augment these outcomes (Merton, 1962). For example, Agirdag et al. (2013) found that teachers’ lower expectations had indirect effects on students’ achievement by triggering an emotional maladaptive scheme. In the present study, principal r qualifications were significant determinants of stronger principal beliefs in their school’s emphasis on academic success and this finding is extremely important given prior evidence on the role of positive expectancies. As suggested by Çoban et al. (2023) teacher’s sense of self-efficacy is directly and indirectly predicted by the principal’s instructional leadership and thus, developing transformational leaders may be a significant objective.

Furthermore, and related to the omnibus finding above, principals with higher qualifications had specifically higher beliefs that their teachers had high expectations about their students’ academic success over and above the generalized belief held by principals. This finding agrees with the work of Agirdag et al. (2013) who also found a positive relationship between teacher expectancies and students’ success but also the work of Jussim (1986) who theorized a causal relationship between the two using a series of steps (see also Darling-Hammond, 1997). Positive expectancies by principals and teachers relate with Rosenthal’s (1974) idea that positive teacher expectancies lead to a supportive and emotionally supportive classroom climate, and optimism, with quality instruction and the provision of positive feedback, and through enhancing a sense of control over academic outcomes (see also McGuigan and Hoy, 2006; Goldstein and Brooks, 2007).

### 5.1 Implications for educational policy and practice

There are several implications of the present study for educational policy. First, recent evidence suggests that one of the most important practices for educational change is professional development (Guskey, 2003; Hattie, 2009; Gaikhorst et al., 2019). Principals are primarily responsible for embedding such activities within schools (Cordingley, 2015) as they are managing teachers. This proposition comes in light of recent empirical findings which demonstrated that principals can create the conditions to successfully implement professional learning communities across cultures (Lee et al., 2016; Hairon and Tan, 2017; Qian and Walker, 2021). Second, principals need to be cognizant of stress levels associated with their job (Prabhakar et al., 2022). For example, Upadyaya et al. (2021) using a person-based analysis reported that 77.3% of the principals belonged to either a “high stress” or “altered stress” profile, with only 22.7% being in the “low stress” category. Beausaert et al. (2021) reported that a salient buffer for a principal’s stress is social capital both internal (bonding) and external (bridging). Related to the sample of this study, educational policymakers in Saudi Arabia should take advantage of the recent regulations that provide both principals and vice principals extra compensation to attract more qualified leaders for schools.

### 5.2 Limitations and future directions

The present study is limited for several reasons. First, the sample size, albeit being associated with adequate levels of power, was modest, to say the least. Second, the study is correlational, and thus, causal inferences about the role of principal qualifications should not be made. Third, a quantitative protocol was employed although the qualitative inquiry has proved to be very informative on the role of principals (e.g., Ryu et al., 2020). Fourth, principals’ effects may be confounded by role uncertainty and ambiguity (Zedeck, 2011) as consensus regarding the role and responsibilities of principals has not been reached. In the future, it is suggested that mixed designs are employed to identify the attitudes and beliefs of principals regarding their schools’ attributes (see also Scheer, 2021). Furthermore, as Qian and Walker (2021) noted principals can create school-based professional learning communities which provide supports that enhance teacher effectiveness (see also Nielsen et al., 2008; Lee and Kim, 2016). Another direction given the pivotal role of principals in influencing their schools’ focus on academics is to examine principals’ well-being (Beausaert et al., 2021) as well as their stress levels along with the necessary resources to mediate those outcomes (Prabhakar et al., 2022).

## Data availability statement

Publicly available datasets were analyzed in this study. This data can be found at: https://timssandpirls.bc.edu/timss2019.

## Ethics statement

The studies involving humans were approved by all ethical procedures as indicated in the Declaration of Helsinki were followed by the TIMSS 2019 international study. The studies were conducted in accordance with the local legislation and institutional requirements. The participants provided their written informed consent to participate in this study. Written informed consent was obtained from the individual(s) for the publication of any potentially identifiable images or data included in this article.

## Author contributions

GS: Conceptualization, Formal analysis, Methodology, Writing – original draft, Writing – review & editing. MA: Data curation, Formal analysis, Funding acquisition, Investigation, Methodology, Writing – review & editing.

## Footnotes

^1^https://timssandpirls.bc.edu/timss2019/
